# Survey to Document the Adverse Reactions After Human Papillomavirus Vaccination Among Japanese Female Youth at a University

**DOI:** 10.1111/jog.70314

**Published:** 2026-05-17

**Authors:** Chigusa Higuchi, Chikako Ogawa, Yoshiaki Iwasaki, Hideharu Hagiya, Hisashi Masuyama

**Affiliations:** ^1^ Educational and Research Management Field, Health Management Department Okayama University Okayama Japan; ^2^ Department of Obstetrics and Gynecology Okayama University Graduate School of Medicine Okayama Japan; ^3^ Department of Infectious Diseases Okayama University Hospital Okayama Japan

**Keywords:** anxiety, human papillomavirus vaccine, Japanese youth, questionnaire, survey

## Abstract

**Aim:**

Concerns about possible adverse events remain a critical barrier in implementing human papillomavirus (HPV) vaccination among Japanese youth. This study aimed to understand the time course of adverse events experienced by HPV vaccine recipients.

**Methods:**

An online questionnaire survey was given to students, faculty, and staff aged 18–26 years, at Okayama University Hospital, who received the HPV vaccine. The survey gathered information on the number of HPV vaccine doses received, prevaccination health conditions, adverse reactions within 2 h and between 2 h and 7 days postvaccination, menstrual irregularities after vaccination, reasons for getting vaccinated, feelings before and after vaccination, and factors providing reassurance during vaccination. Prevalence of symptoms was expressed as numbers and percentages, and analyses were performed using Chi‐squared or Fisher's exact tests.

**Results:**

Responses were obtained from 299 participants, yielding a 75% response rate. Approximately 60% participants reported local pain, 30% swelling, and 4% fever. Most symptoms resolved on the vaccination day itself or the following day, although some persisted for 3–7 days. Over 80% participants rated their pain between 0 and 3 on numerical rating scale of 0–10. While 60% experienced anxiety before vaccination, 90% reported no anxiety afterward.

**Conclusions:**

Our study presents one of the first comprehensive accounts of post‐HPV vaccination adverse events and their time course, and underpins the importance of disseminating detailed information about vaccine‐associated adverse reactions to encourage greater vaccine uptake.

## 1 Introduction

In Japan, approximately 10 000 women are diagnosed with invasive cervical cancer (CC) annually, and 3000 women die of the disease each year. Notably, there has been no decline in these numbers for approximately 30 years [1, 2]. In contrast to the decreasing trend in the incidence of CC in other high‐resource countries, in Japan, its incidence—particularly among women of reproductive age—and mortality rates among young and middle‐aged women due to CC are increasing [3]. The human papillomavirus (HPV) vaccine prevents HPV infection and has been proven to be effective in reducing CC risk. The World Health Organization (WHO) aims to eliminate CC worldwide by implementing three key strategies: 90% of girls fully vaccinated with the HPV vaccine by the age of 15; 70% of women screened using a high‐performance test by the age of 35, and again by the age of 45, and 90% of women with precancer treated and 90% of women with invasive cancer managed [4].

In Japan, a publicly funded HPV vaccination program for girls from the sixth grade of elementary school to the first year of high school was introduced in April 2013. However, following reports of adverse reactions, active recommendations were suspended in June of the same year. As evidence confirmed the vaccine's safety and effectiveness in preventing CC, active recommendations for routine vaccination were reinstated in April 2022. Additionally, a 3‐year catch‐up vaccination program was launched for women born between 1997 and 2008, who had missed the opportunity to receive the vaccine. Despite these efforts, vaccination rates remain insufficient, and the catch‐up program has been extended until the end of March 2026 [5, 6].

Despite the suspension of HPV vaccination recommendation in Japan by the Japanese Ministry of Health, Labor and Welfare (MHLW) in 2013, an increase in the number of vaccines delivered monthly to healthcare facilities has increased from 878 doses between December 2016 and April 2017 to 35 396 doses from January to March 2021 [7]. This trend reflects efforts of the Japanese Government to convey information about HPV vaccination. Despite the initiative, participation in the catch‐up program remains insufficient, putting many individuals at risk of missing the opportunity for vaccination [8]. Overall, although the number of vaccinations increased following the resumption of active recommendations in 2022, achieving the WHO's 90% vaccination coverage target for CC prevention remains a significant challenge.

Although a questionnaire‐based study in Nagoya City showed no causal association between HPV vaccines and reported symptoms [9], there remains an information gap on the incidence of HPV vaccine‐related adverse events in Japan. According to a recent study, concerns regarding possible adverse events have been high among 47.4% healthcare students [10]. Furthermore, immunization stress‐related responses (ISRR) are recognized obstacles to a country's immunization program [11].

As a solution, providing accurate information on the preventive effects of the HPV vaccine against infection and CC, along with detailed data on common adverse reactions and their duration, can help respond to the ISSR. In this study, we aimed to analyze the progression and characteristics of adverse reactions following catch‐up HPV vaccination in the Japanese female population at the Health Service Center of Okayama University using survey data. We asked participants, irrespective of the type of HPV vaccine they received, not only about the occurrence or nonoccurrence of adverse reactions but also about the timing of appearance and disappearance of symptoms, because a better understanding of the time course might contribute to addressing and alleviating the anxiety pre‐ and postvaccination. We conducted this survey to promote understanding of correct information on HPV vaccines by providing information to the general public and feedback to students and faculty members of Okayama University.

## 2 Methods

### 2.1 Study Design and Population

An online questionnaire survey was administered at the Okayama University Hospital to evaluate the frequency of adverse reactions to the HPV vaccine. This study targeted to enroll 400 female students and female medical and nonmedical staff and faculty members, aged 18–26 years, of Okayama University who received the HPV vaccine (Silgard9) at the mass vaccination site at Okayama University Hospital between August 2023 and January 2024. Mass vaccination included nonmedical faculty members, and students was conducted over 3 days on August 7–9, 2023; October 16–18, 2023; and January 22–24, 2024.

One week after vaccination, the participants were invited via email to complete a web‐based questionnaire assessing the presence and duration of symptoms, irrespective of the type of vaccine taken against HPV. All respondents received a 500‐yen gift card as compensation (equivalent to approximately $3.55 USD as of January 2024 exchange rate of 1 USD = 140.94 yen). Because the participants belonged to a nearly identical group in terms of age, sex, and other factors, no selection bias was assumed.

### 2.2 Ethics Statement

The study protocol was reviewed and approved by the Ethics Committee of Okayama University Graduate School of Medicine, Dentistry, and Pharmaceutical Sciences and Okayama University Hospital (approval number: 2312‐001). All the participants provided informed consent through a web‐based questionnaire to take part in the study and publish the results. Only those who provided consent were included.

The study results were previously presented at the 76th Annual Congress of Japan Society of Obstetrics and Gynecology that was held in Yokohama, Japan, on 19–21 April 2024, presented at the 54th Chugoku‐Shikoku University Health Management Research Conference that was held in Kochi, Japan, on 22–23 August 2024, and presented at the IPVC2024, the 35th Annual Conference of the International Papillomavirus Society, on November 12–15, 2024. A summary of this study, including selected figures, was previously disseminated in an institutional press release related to the conference presentation.

### 2.3 Survey

The survey was administered as a web‐based questionnaire using Moodle. It was conducted in both Japanese and English and included questions on the respondents' attributes (age group and status) and body condition, such as underweight or overweight status, susceptibility to dizziness, menstruation‐related symptoms, abdominal pain, and ongoing medical conditions. Additionally, it gathered information on the number of HPV vaccine doses received, prevaccination health conditions, adverse reactions within 2 h postvaccination, adverse reactions between 2 h and 7 days postvaccination, menstrual irregularities after vaccination, reasons for deciding to get vaccinated, feelings before and after vaccination, and factors that provided reassurance during vaccination.

### 2.4 Assessment of Adverse Events

For adverse reactions occurring between 2 h and 7 days after vaccination, the survey assessed local reactions such as swelling, redness, pain, and itching, as well as systemic reactions, including fever ≥ 37.5°C, sense of fatigue, dizziness, nausea, muscle soreness, joint pain, and rash. Participants were asked to indicate whether they experienced each symptom, and if they did, they were required to select the last day they observed the symptoms.

### 2.5 Statistical Analyses

Statistical analyses were conducted using SPSS software (version 26; IBM, Armonk, New York, USA). The prevalence of symptoms is expressed as numbers and percentages, and analyses were performed using the Chi‐squared test or Fisher's exact test. Statistical significance was set at *p* ≤ 0.05.

## 3 Results

Between August 14, 2023, and February 6, 2024, 299 participants completed the survey, resulting in a valid response rate of 75%. The data from valid responses is presented in Appendix I. Among respondents, 171 (57.2%) were nonmedical faculty members and students. The participants were required to share their personal details, age, occupation, body mass index (BMI), underlying disease, and physical conditions before vaccination. The participant characteristics are presented in Table 1. Among them, 111, 102, and 86 patients received the first, second, and third doses, respectively.

**TABLE 1:** Characteristics of the survey participants.

		Adverse reactions (*n* = 224)		No adverse reaction (*n* = 75)		Total (*n* = 299)	*p*
First dose		79	35.3%	32	42.7%	111	
Second dose		77	34.4%	25	33.3%	102	
Third dose		68	30.4%	18	24.0%	86	*p* = 0.203
Age (y)	10s	29	12.9%	15	20.0%	44	
	20s	195	87.1%	60	80.0%	255	*p* = 0.203
Student		214	95.5%	75	100.0%	289	
Staff or faculty		10	4.5%	0	0.0%	10	*p* = 0.098
Body condition	BMI under 18.5	30	13.4%	8	10.7%	38	*p* = 0.541
	BMI over 30	5	2.2%	2	2.7%	7	*p* = 0.830
	Prone to dizziness	33	14.7%	10	13.3%	43	*p* = 0.766
	Fainted in the past	20	8.9%	2	2.7%	22	*p* = 0.073
	Prone to stomach aches	19	8.5%	7	9.3%	26	*p* = 0.822
	Menstrual‐related symptoms	44	19.6%	16	21.3%	60	*p* = 0.753
	Allergic disease	32	14.3%	7	9.3%	39	*p* = 0.272
	Other underlying disease	1	0.4%	1	1.3%	2	*p* = 0.416

*Note:* Chi‐squared test or Fisher's exact test were used. A *p*‐value of ≤ 0.05 is considered statistically significant.

The participants were categorized based on the presence or absence of adverse reactions. The study included 44 teenagers (14.7%) and 255 individuals in their twenties (85.3%), all of whom were female. Students comprised the majority, accounting for 289 participants (96.7%). Regarding underlying health conditions, the results showed that 60 participants (20.1%) had menstrual‐related symptoms, 43 (14.4%) were prone to dizziness, 39 (13.0%) had allergic diseases, 38 (12.7%) had a BMI of 18.5 or lower, 26 (8.7%) were prone to abdominal pain, and 22 (7.4%) fainted in the past. Although no statistically significant association was found between adverse reactions and body constitution, the participants who had fainted in the past or had allergic diseases tended to exhibit a higher incidence of adverse reactions.

**FIGURE 1:**
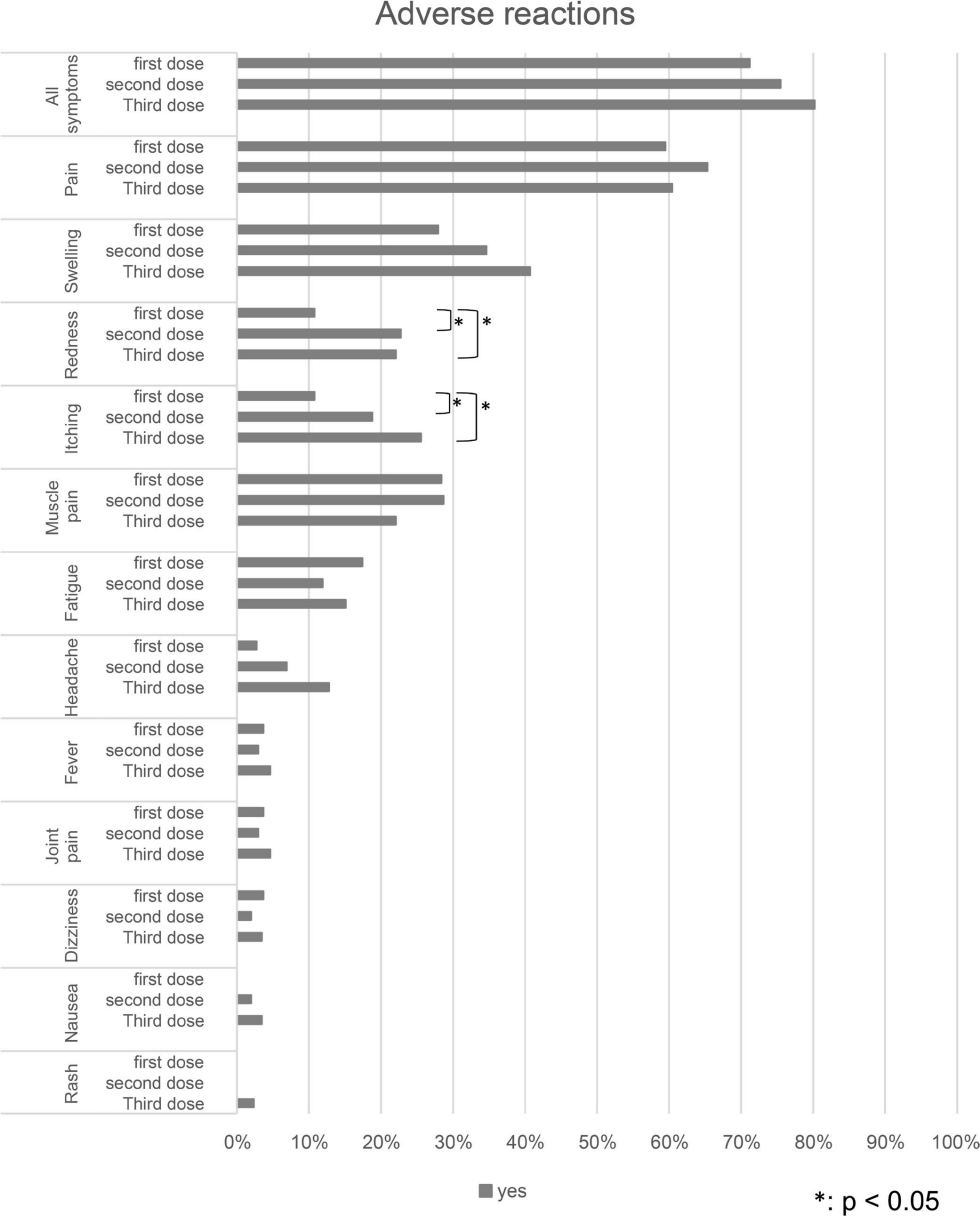
Presence of adverse reactions. Participants were asked to mention the symptoms they experienced after vaccination. A *p* value ≤ 0.05 was considered statistically significant.

**FIGURE 2:**
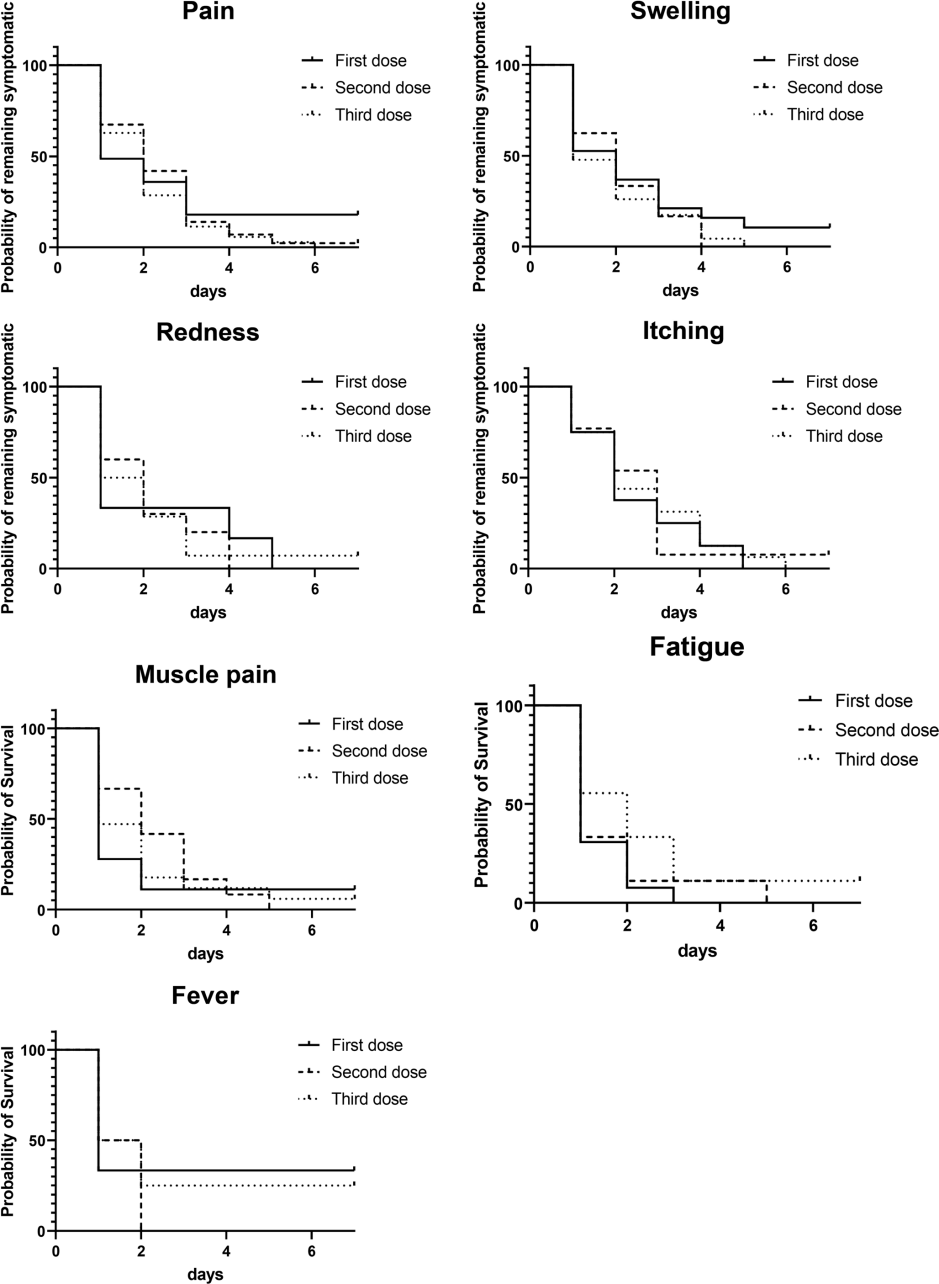
Kaplan–Meier curve showing symptom persistence in participants with symptoms at baseline. Participants were asked to mention the local reactions (swelling, redness, pain, itching) and systemic symptoms (fever ≥ 37.5°C, sense of fatigue, muscle soreness) after HPV vaccination and the last date when they experienced the symptoms. Participants who were asymptomatic at baseline were excluded from the Kaplan–Meier curve analysis.

Figure 1 illustrates the occurrence or nonoccurrence of adverse reactions, and Figure 2 presents a Kaplan–Meier curve indicating the day symptoms resolved for those who experienced symptoms. In total, 224 participants (74.9%) experienced at least one adverse reaction, such as pain, redness, swelling, or a sense of fatigue. Among them, 79, 77, and 68 participants reported adverse reactions to the first, second, and third doses, respectively. Adverse reactions observed within 2 h of vaccination included pain, swelling, and fever. However, none of the participants experienced symptoms requiring medical intervention, such as vasovagal reflex or anaphylactic shock. In most cases, the symptoms disappeared within the first 2 days.

Pain was the most commonly reported adverse reaction, affecting 66 participants (59.5%) after the first, 66 participants (65.3%) after the second, and 52 participants (60.5%) after the third dose. Regarding pain resolution, > 70% of participants who received the first dose reported that their pain subsided within the same or the following day. In contrast, for the second and third doses, only approximately 50% experienced pain resolution within this timeframe, with a higher proportion reporting pain lasting 2–3 days postvaccination. Pain persisted beyond Day 7 in seven participants after the first dose, and in one participant after the second dose.

**FIGURE 3:**
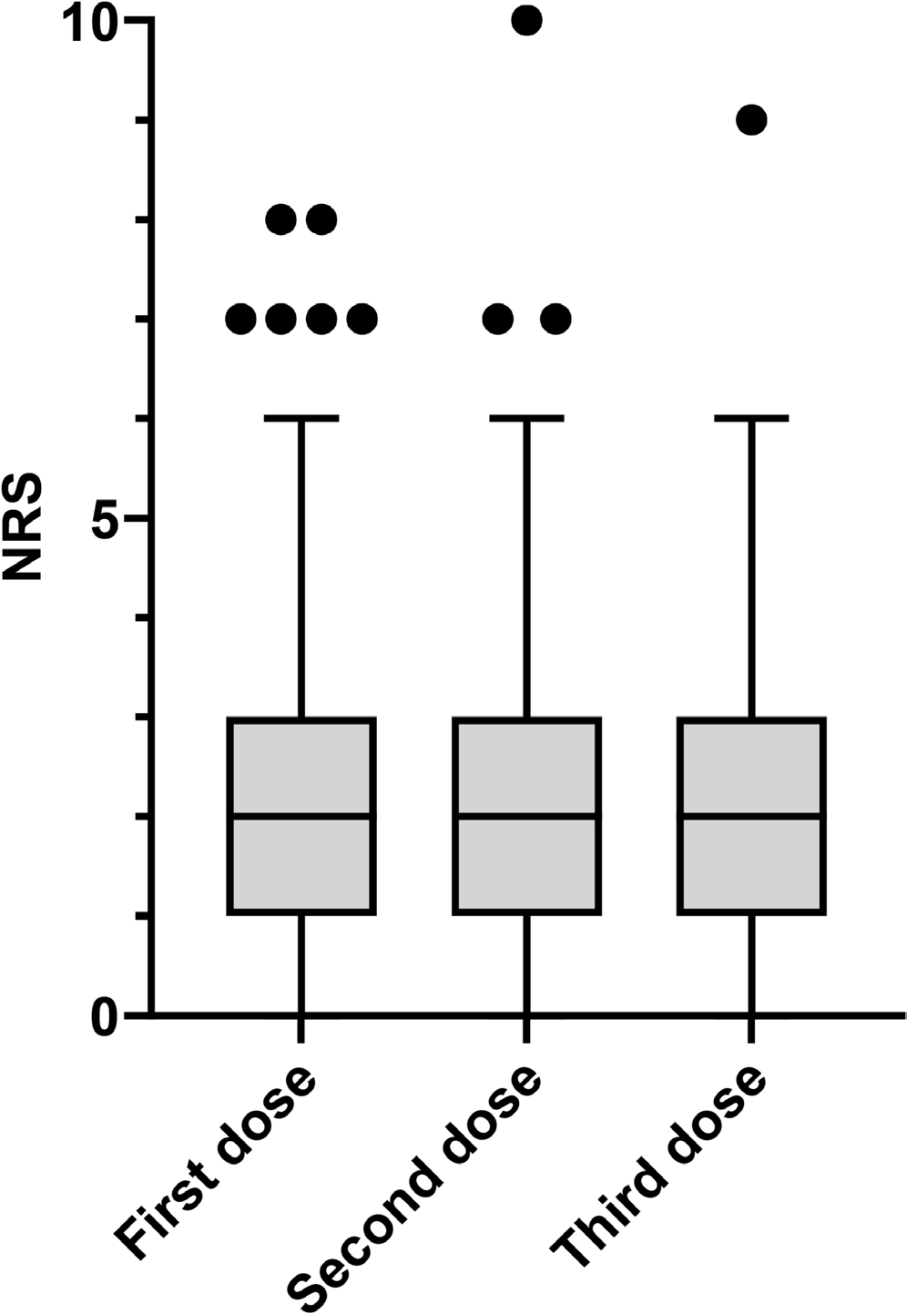
Perceived severity of pain scored on a numerical rating scale. Participants were asked to rate their post‐HPV vaccination pain at the point of greatest pain after HPV vaccination on a scale using a score of “0” for no pain at all and “10” for the worst pain.

A comparison of the first, second, and third doses revealed significant differences in the incidence of redness and itching. These symptoms were reported more frequently after the second and third doses than after the first dose. The median numerical rating scale score was 2 for all doses, suggesting the rarity of severe pain (Figure 3).

In total, nine participants experienced fever at least once after the first, second, or third doses, with fever occurring 11 times in total out of 297 vaccinations (3.7%). Table S1 shows the presence or absence of fever and other symptoms observed after each vaccination. Fever was reported in four, three, and four participants after the first, second, and third doses, respectively. Additionally, two participants (Cases 1 and 2) experienced fever twice during their vaccinations. However, none of the participants reported fever after all the three doses. We analyzed the relationship between fever occurrence and factors including participant attributes, underlying conditions, prevaccination health status, meals, and sleep. However, no significant correlations were observed.

Seven participants after the first dose, eight after the second dose, and six after the third dose reported that their postvaccination menstrual cycles differed from their usual cycles (Table S2). Additionally, when asked how many cycles their menstrual irregularities had persisted, only one participant (after the second dose) reported experiencing two consecutive irregular cycles. Most participants stated that they had experienced irregular menstruation once and that their next period had not yet occurred at the time of the survey.

**FIGURE 4:**
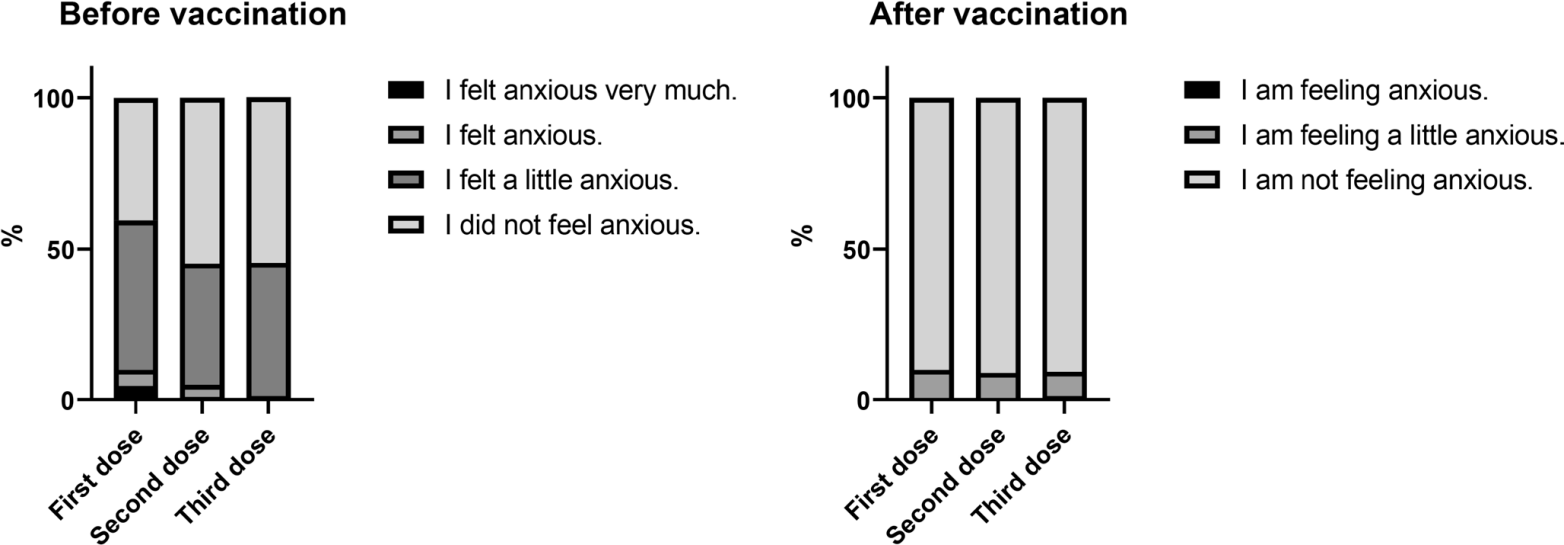
Various levels of anxiety experienced by the percentage of participants before and after vaccination. Participants were asked to select the option that was closest to how they felt before and 1 week after HPV vaccination.

The percentage of participants who felt anxious before vaccination was 57.9% before the first dose, 44.6% before the second dose, and 45.3% before the third dose. In contrast, the percentage of participants who reported feeling anxious 1‐week postvaccination decreased to 9.3% after the first dose, 8.9% after the second dose, and 9.6% after the third dose (Figure 4). Open‐ended responses on reasons due to which participants felt anxious were categorized through postcoding and are presented in Table 2. The most common concern was related to adverse reactions (survey question 26, Appendix II), reported by 119 participants (89%). The most frequently mentioned concern was worry about adverse reactions, reported by 59 participants (43%). Additionally, 23 participants (17%) were worried about experiencing serious adverse reactions, 15 (11%) reported anxiety due to uncertainty about what kind of adverse reactions might occur, and nine (7%) mentioned concerns influenced by media reports about serious adverse reactions.

**TABLE 2:** Number of participants who experienced various types of concerns before vaccination.

		*n*
Adverse reactions	Adverse reactions only	59
	Serious adverse reactions	21
	I do not know what kind of adverse reactions I will have	13
	Pain	9
	I saw reports of serious adverse reactions	9
	Because I live alone	2
	Comparison with adverse reactions to COVID‐19 vaccine	5
	Comparison with adverse reactions to influenza vaccine	1
Others	Anxiety about cervical cancer	1
	Wondering if the vaccine will be effective	2
	Worried if already infected with HPV	2
	About sexual activity	1
	Dislike injections	4
	Parental disapproval	2
	Safety	2

*Note:* Survey question 26: If you felt anxious before vaccination, please describe your concern.

In response to the question on the factor that made the respondents feel comfortable or reassured during the vaccination process, the largest number of respondents indicated that they would feel safer if they were inoculated at Okayama University Hospital (Table 3). Many of the respondents in the free‐response section said they were glad to have the inoculation at Okayama University, suggesting that providing the opportunity for inoculation at the university where they are enrolled would make them feel more reassured.

**TABLE 3:** Factors that provided reassurance during vaccination.

Reassuring factors	First dose	Second dose	Third dose	Total dose
I can receive the vaccine with everyone	32	24	22	78
I can receive the vaccine at Okayama University Hospital	92	89	80	261
I can consult a gynecologist	30	21	16	67
The brochure I received	29	15	14	58
Shuttle bus service	32	20	16	68
Information from the Health Service Center	49	26	21	96
Information provided by municipalities	13	10	8	31
Others	1	1	1	3

*Note:* Survey questions 30 and 31: Is there anything that made you feel comfortable or reassured during the vaccination process?

## 4 Discussion

In this study, we comprehensively investigated and analyzed postvaccination adverse reactions among all female faculty members and students who received the HPV catch‐up vaccination at Okayama University Hospital.

Over the last decade, vaccine hesitancy has attracted considerable attention. Reports have indicated that the assessment of adverse events by the general population differs considerably from that by health authorities. Having a direct or indirect experience of adverse events negatively influences attitudes toward vaccination, resulting in more frequent and intense experiences with adverse events [12]. Therefore, prospective survey results of a cohort vaccinated in a supportive environment, as in the present study, may capture postvaccination symptoms more accurately.

The most frequently reported local and systemic adverse reactions after the four‐valent or nine‐valent HPV vaccine in girls aged 9 through 15 years were pain (89.3%), swelling (47.8%), and erythema (34.1%) at the injection site, and headache (14.6%) [2]. We found that 70%–80% experienced some form of adverse reactions following HPV vaccination. However, most reactions were mild, with localized pain, and the symptoms resolved within 2–3 days.

The follow‐up survey was conducted 1 week after vaccination. This timing was set based on prior studies indicating that most postvaccination adverse reactions appear and resolve within one to several days after vaccination [13, 14]. The goal was to survey while recipients' memories were still fresh.

However, adverse reactions to the HPV vaccine persisting for more than 1 week after vaccination or appearing more than a week later have been documented. Therefore, it cannot be denied that the timing of this survey may have missed some adverse reactions. In this survey, for respondents who had not yet resolved symptoms that appeared after vaccination at the time of their initial response, follow‐up confirmation of their subsequent symptom status was conducted individually via email or in person. Additionally, during the next vaccination visit, confirmation was made in person regarding the occurrence of any adverse reactions more than 1 week after vaccination.

In Japan, several factors contribute to the low HPV vaccination uptake, including concerns about adverse reactions, insufficient information about the vaccine, and the perception that vaccination is inconvenient. Postmarketing surveillance of adverse reactions has been conducted in many countries, including Japan. Although no serious safety concerns have been identified, studies indicate that pain is a common reaction following vaccination. However, detailed information regarding the typical duration of symptoms after vaccination remains limited.

Although there have been reports on the frequency of local pain, no data exists on its intensity or duration. From our data, even when pain occurs, it is typical for pain to last for 2–3 days at a score of three points or less. Pain that is too strong to bear or lasts for 7 days or more is atypical, and it may be worth considering a visit to the hospital.

In this study, only vaccinated individuals were analyzed. The results revealed that more than half of the participants felt anxious before receiving their first dose. Although the proportion of individuals with strong anxiety decreased after the second and third doses, over 40% still reported feeling anxious. These results suggest that concerns about the HPV vaccine persist in the Japanese population. Open‐ended responses revealed that most concerns were related to adverse reactions, with participants expressing worries about experiencing serious adverse reactions or uncertainty about the kind of adverse reactions that might occur. Additionally, some participants stated, “I looked up general adverse reactions to the HPV vaccine, but I could not find clear information.” Over the past decade (including the survey period), the MHLW, as well as academic institutions, have continuously disseminated substantial information regarding HPV vaccination. However, there may be challenges in ensuring that people who need this information can easily access it^6^. Furthermore, even when information is obtained, it does not necessarily lead to actual behavioral change [15]. These represent significant issues for promoting HPV vaccination in Japan.

Among all the vaccine recipients, the overall incidence of fever was 3.7% (11 out of 297 vaccinations). In contrast, among those who had previously experienced fever, the likelihood of developing fever again at subsequent doses was 25% (two out of eight vaccinations). These findings suggest that although the risk of fever slightly increases after an initial occurrence, it does not necessarily occur with every subsequent dose. These findings suggest that individual characteristics may influence the occurrence of fever as a postvaccination adverse reaction. However, this study could not determine whether other factors, such as health conditions on the day of vaccination, played a role. Nevertheless, the fact that fever does not occur every time in the same individual provides valuable information for those who have experienced postvaccination fever at least once.

In our study, no consistent menstrual changes were observed following the HPV vaccination. In cohort studies, symptom variation is present between cohorts, including menstrual abnormalities, and the impact of vaccines on menstruation remains inconclusive. However, since this study was conducted 1‐week postvaccination, the timing may have been too early to assess menstrual irregularities accurately.

A previous study by Liu et al. [16] showed that the public's perception of the HPV vaccine fluctuated, mirroring government policy decisions and media coverage. The suspension of HPV vaccine recommendations by the Japanese Government was a key moment that led to a sharp decline in public trust, which can be attributed to increased uncertainty and fear regarding vaccine safety. Apart from the fear of adverse events, parental disapproval of vaccination and taboo against sexually transmitted diseases served as critical barriers to HPV vaccination [17]. Our findings also indicate the presence of such factors.

However, with the resurgence of advocacy in 2020, public confidence improved, which likely influenced efforts to reinstate vaccine recommendations. The latest report shows high HPV infection awareness boosted vaccination intentions among students (50.5%) and mothers (38.8%) [18]. Stronger CC control measures are needed to plateau approximately one‐half of the WHO target values by 2028, especially for the vaccine‐resumed generation [19]. Our findings address a critical gap that encompasses concerns about HPV vaccination safety, one of the most commonly reported barriers for young people aged 9–26 years [17].

This study has some limitations. The frequency of reported adverse reactions may have been over‐ or under‐estimated in this self‐reported survey. The reported adverse events and comorbidity histories could not be verified. We considered only short‐term adverse effects, and long‐term monitoring is required. Furthermore, since this study involved repeated surveys within the same group, participants may have become more aware of symptoms such as redness, itching, and headaches after completing the first survey. Consequently, they may have paid closer attention to these symptoms during the administration of their second and third doses. Therefore, the possibility of a bias that may have influenced the findings cannot be ruled out. Potential bias also exists due to the small scale and single‐center study design. Japan's routine vaccination program targets only females; consequently, this survey's focus on females alone is also an inherent limitation. The questionnaire used in this survey was not prevalidated, and the lack of evidence supporting the survey's reliability and validity is a limitation of this study. Finally, compensation offered to the respondents might have induced risks of response and performance biases.

Nonetheless, despite these limitations, our study presents one of the first comprehensive accounts of adverse events that appear post‐HPV vaccination, as well as the severity and frequency of their appearance, progression, and resolution over time. In a society where concerns about vaccines persist, even mild symptoms can cause significant anxiety among recipients. Several previous studies and systematic reviews have reported no evidence of vaccine‐related serious adverse events [20, 21, 22]. Fever and local pain are commonly observed, whereas the profiles of other medical conditions have been shown to be comparable between vaccinated and unvaccinated populations. Although these studies are large in scale, they are primarily based on reports of serious adverse events. Therefore, information regarding the clinical course and resolution of postvaccination symptoms remains limited. Such data alone are not sufficient to alleviate anxiety, as insights into how symptoms evolve and resolve remain unclear. The present survey allows individuals to visualize and resolve their symptoms.

This post‐HPV‐vaccination survey among students and staff at Okayama University Hospital revealed that 70%–80% of participants experienced some form of adverse reaction following HPV vaccination. However, most reactions were mild with localized pain, and the symptoms resolved within 2–3 days. The findings suggested that while the risk of fever slightly increased after an initial occurrence, it did not necessarily occur with every subsequent dose. These adverse events and their time course underscore the importance of disseminating detailed information about vaccine‐associated adverse reactions to encourage greater HPV‐vaccine uptake.

## Author Contributions

**Chigusa Higuchi:** conceptualization, methodology, investigation, data curation, writing – original draft, supervision. **Chikako Ogawa:** conceptualization, methodology, writing – review and editing, project administration, funding acquisition. **Yoshiaki Iwasaki:** conceptualization, methodology, investigation. **Hideharu Hagiya:** conceptualization, investigation. **Hisashi Masuyama:** conceptualization.

## Funding

This work was supported by Ministry of Health, Labour and Welfare (MHLW) under the HPV Vaccination Hub Hospital Development Project, 0401‐10.

## Disclosure

The study results were previously presented at the 76th Annual Congress of Japan Society of Obstetrics and Gynecology that was held in Yokohama, Japan, on 19–21 April 2024, presented at the 54th Chugoku‐Shikoku University Health Management Research Conference that was held in Kochi, Japan, on 22–23 August 2024, and presented at the IPVC2024, the 35th Annual Conference of the International Papillomavirus Society, on November 12–15, 2024. A summary of this study, including selected figures, was previously disseminated in an institutional press release related to the conference presentation.

## Ethics Statement

The study protocol was reviewed and approved by the Ethics Committee of Okayama University Graduate School of Medicine, Dentistry, and Pharmaceutical Sciences and Okayama University Hospital (approval number: 2312‐001).

## Consent

All the participants provided informed consent through a web‐based questionnaire to take part in the study and publish the results. Only those who provided consent were included.

## Conflicts of Interest

The authors declare no conflicts of interest.

## Supporting information


**Appendix I.** Survey result dataset.

**Appendix II.** Survey questionnaire.

**Table S1:** Cases with at least one episode of fever over 37.5°C.
**Table S2:** Deviation in the time period from the usual period date after the administration of human papillomavirus vaccination.

## Data Availability

The data that supports the findings of this study are available in the Supporting Information of this article.
