# Supplementary material for: Survey to Document the Adverse Reactions After Human Papillomavirus Vaccination Among Japanese Female Youth at a University
Source: J Obstet Gynaecol Res. 2026 May 17;52:e70314. doi: 10.1111/jog.70314 (PMC13180500; doi:10.1111/jog.70314)
Supplement: Supplementary file 2 — Appendix II. Survey questionnaire. [file JOG-52-0-s002.docx]

**Post-vaccination survey of HPV**

**(One week after vaccination)**

This survey is to evaluate the frequency of adverse reactions to HPV vaccine at Okayama University Hospital. The Health Service Center of Okayama University is conducting this survey in order to promote under standing correct information to HPV vaccine through providing information to the general public and feedback to student s and faculty of Okayama University. Please answer this survey about one week after the vaccination, regardless of whether adverse reactions have occurred or not. Thank you for your cooperation.

The results of this survey will be compiled and published in a form that is not ident ify individuals. The response rate of the survey is very important to obtain accurate results so please answer it. Thank you in advance.

1. If you understand the purpose of this survey and agree to answer, please proceed with the survey.

- I agree with this survey and answer the questionnaire.
- I do not agree with this survey.

1. Please select the date of your vaccination.

- yyyy/mm/dd

1. How many times have you been vaccinated against HPV this time?

Note: It does not matter what type of vaccine against HPV.

E.g.) If you have been vaccinated Cervarix twice in the past and vaccinated Gardasil this time, please select 'third time'

- First time
- Second time
- Third time

Please tell us about yourself.

1. Your age group

- Teens
- 20-29
- 30 and over

1. Your occupation

- Student
- Medical staff
- Staff (except medical staff)
- Medical faculty
- Faculty (except medi cal faculty)
- Others

1. Please select all that you apply.

- None of the followings
- Thin (BMI: under 18.5 ）
- Overweight (BMI: over 30)
- I tend to get lightheadedness.
- I have fainted in the past.
- I tend to get stomachaches.
- I have severe mens trual- related symptoms, such as period pain and premens trual syndrome.
- I have an allergic disease.
- I have other underlying diseases.

1. If you selected 'I have other under lying disease.', please describe the name of disease.

Please tell us your situation before vaccination.

1. Please tell us your physical condition before vaccination.

- As usual
- Worse than usual

1. If you selected 'Worse than usual', please describe concrete symptom.
2. Please tell us your sleep before vaccination.

- I slept better than usual.
- I slept as usual.
- I slept less than usual.

1. Please tell us your meal before vaccination.

- I had both breakfast and lunch on the day.
- I had only breakfast on the day.
- I had only lunch on the day.
- I did not have both breakfast and lunch.

Please tell us the symptoms after vaccination.

1. Did you feel sick within 2 hours after HPV vaccination?

- Yes
- No

1. What symptoms have appeared?

- Pain
- Fever
- Itching
- Rash
- Nausea
- Vomiting
- Diarrhea
- Dyspnea
- Decreased blood pressure
- Loss of consciousness
- Others

1. If you selected 'Others', please describe the symptoms.

Please tell us the adverse reactions after 2 hours after vaccination.

1. What is the local reaction at the vaccination site after HPV vaccination and when did it appear? Please select the last date you had symptoms.

|  | None | On the vaccination day | The day after vaccination | 2 days later | 3 days later | 4 days later | 5 days later | 6 days later | After 7 days |
| --- | --- | --- | --- | --- | --- | --- | --- | --- | --- |
| Swelling |  |  |  |  |  |  |  |  |  |
| Redness |  |  |  |  |  |  |  |  |  |
| Pain |  |  |  |  |  |  |  |  |  |
| Itching |  |  |  |  |  |  |  |  |  |

1. What are systemic symptoms after HPV vaccination and when did they appear? Please select the last date you had symptoms.

|  | None | On the vaccination day | The day after vaccination | 2 days later | 3 days later | 4 days later | 5 days later | 6 days later | After 7 days |
| --- | --- | --- | --- | --- | --- | --- | --- | --- | --- |
| Fever 37.5 ℃ and over |  |  |  |  |  |  |  |  |  |
| Headache |  |  |  |  |  |  |  |  |  |
| Sens e of fatigue |  |  |  |  |  |  |  |  |  |
| Dizzy |  |  |  |  |  |  |  |  |  |
| Nausea |  |  |  |  |  |  |  |  |  |
| Muscle soreness |  |  |  |  |  |  |  |  |  |
| Joint pain |  |  |  |  |  |  |  |  |  |
| Rash |  |  |  |  |  |  |  |  |  |

1. Please tell us about your post-HPV vaccination pain.

Using a score of "0" for no pain at all and a score of "10" for the worst pain you could think of and imagine, what was your score at the point of greatest pain after HPV vaccination?

0 1 2 3 4 5 6 7 8 9 10

Please tell us about period after HPV vaccination.

1. Did your period after the HPV vaccination differ from your usual period? If you were vaccinated the second or later dose this time, please include your period after the previous HPV vaccination.

- As usual
- My last period was different from my usual one.
- I do not know because next period has not come yet.

1. For those who selected 'My last period was different from my usual one.', what symptoms did you recognize?

- Period ﬂow increased.
- Period ﬂow decreased.
- Period became earlier.
- Period delayed.
- Period last became longer.
- I had severe period pain.
- I had sever headache during period.
- Others

1. If you selected 'Others', please describe what differences you found.
2. How many times did period different from usual period?

- I had one time and next one was as usual.
- I had one time and next one has not come yet.
- I had two times and third one was normal.
- I had two times and third one has not come yet.
- More than 3 times

1. If you had any other adverse reactions except for question no.15 and no.16 after 2 hours after HPV vaccination, please tell us what symptoms you had.
2. How do you feel about the adverse reactions after HPV vaccination? Please compare the answer to the adverse reactions after COVID-19 vaccination.

- It was milder than the adverse reactions of COVID-19 vaccination.
- It was slightly milder than the adverse reactions of COVID-19 vaccination.
- It was no differences between HPV vaccination and COVID-19 vaccination.
- It was slightly more severe than the adverse reactions of COVID-19 vaccination.
- It was more severe than the adverse reactions of COVID-19 vaccination.
- I did not receive COVID-19 vaccine.

Please tell us your thoughts on the HPV vaccine and cervical cancer.

1. What was the reason for your decision to get the HPV vaccine?

- It can prevent HPV infection.
- It can prevent cervical cancer.
- My parents recommended it.
- My friends and others recommended it.
- My doctor or other medical professional recommended it.
- My friends already vaccinated.
- I understood about adverse reactions and safety.
- The vaccination is free.
- I answered previous HPV vaccination survey.
- No particular reason.

1. Please select the one that is most closest match to how you felt before HPV vaccination.

- I felt anxious very much.
- I felt anxious.
- I felt a little anxious.
- I did not feel anxious.

1. If you felt anxious before vaccination, please describe what your concern is.
2. Please select the one that is most closest match to how you feel now. (About 1 week passed)

- I am feeling anxious very much.
- I am feeling anxious.
- I am feeling a little anxious.
- I am not feeling anxious.

1. If you are feeling anxious, please describe what your concern is.
2. After HPV vaccination, how much would you like to recommend HPV vaccination to your friends ?

- I would like to strongly recommend.
- I would like to recommend it.
- Neither
- I would not like to recommend.
- I will not recommend at all.

1. Was there anything that made you feel comfortable or reassured during the vaccination process?

- I can receive the vaccine with everyone.
- I can receive the vaccine at Okayama University Hospital.
- I can consult a gynecologist.
- The brochure I received.
- Shuttle Bus Service
- Information from the Health Service Center.
- Information provided by municipalities.
- Others

1. If you selected 'Others', please describe what helped reduce your anxiety.
2. Do you have any other comments or feedback?
3. Once these HPV vaccine survey data from the August 2023 to January 2024 has been compiled, it will be published on the Health Service Center's website. We can send to those who wish to be received an ema il about notify of the publication. Would you like to be notiﬁed by ema il?

- Yes
- No
