# Supplementary material for: Survey to Document the Adverse Reactions After Human Papillomavirus Vaccination Among Japanese Female Youth at a University
Source: J Obstet Gynaecol Res. 2026 May 17;52:e70314. doi: 10.1111/jog.70314 (PMC13180500; doi:10.1111/jog.70314)
Supplement: Supplementary file 3 — Table S1: Cases with at least one episode of fever over 37.5°C. Table S2: Deviation in the time period from the usual period date after the administration of human papillomavirus vaccination. [file JOG-52-0-s001.docx]

**Supporting Information**

**Table S1.** Cases with at least one episode of fever over 37.5 ℃


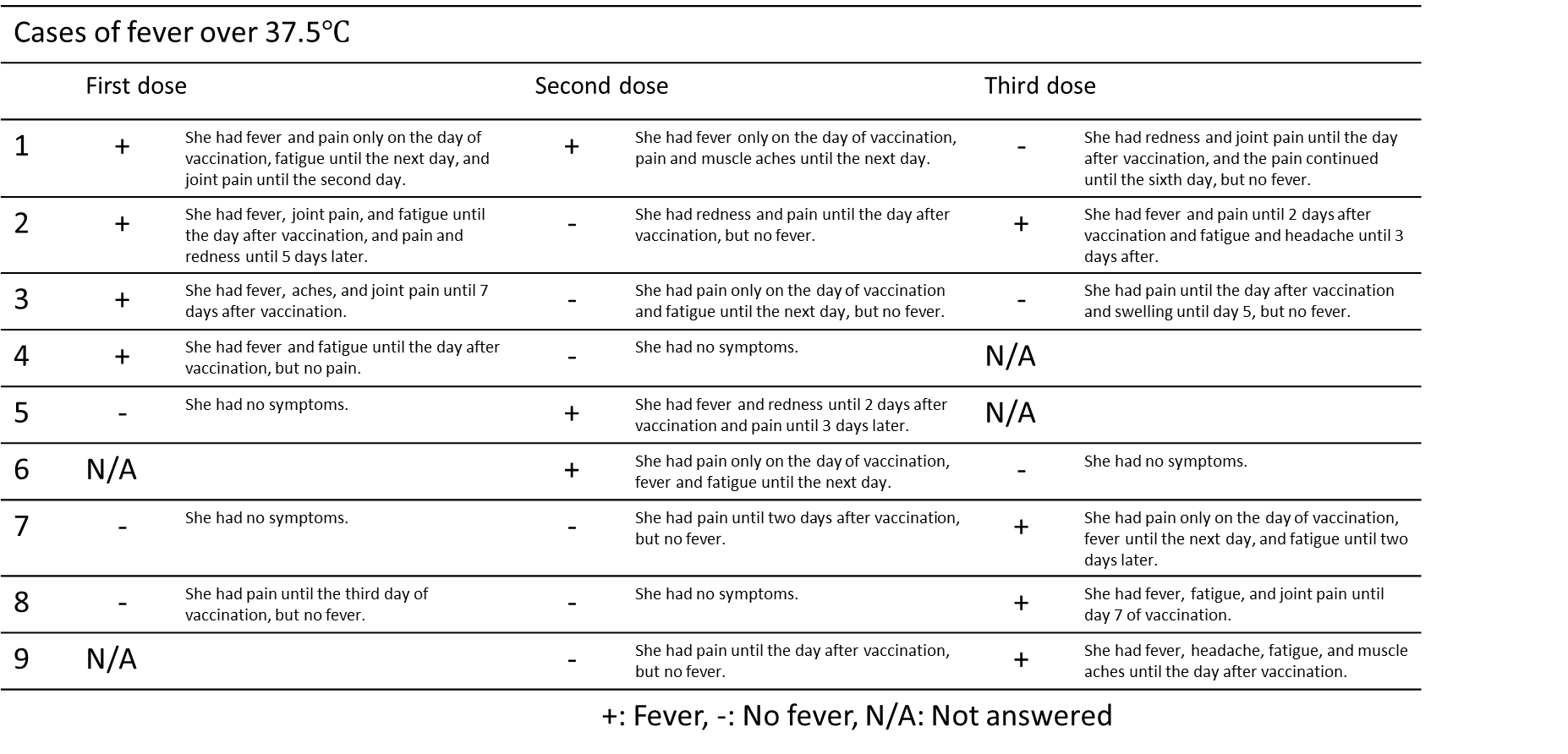


**Table S2.** Deviation in the time of period from the usual period date after the administration of human papillomavirus vaccination


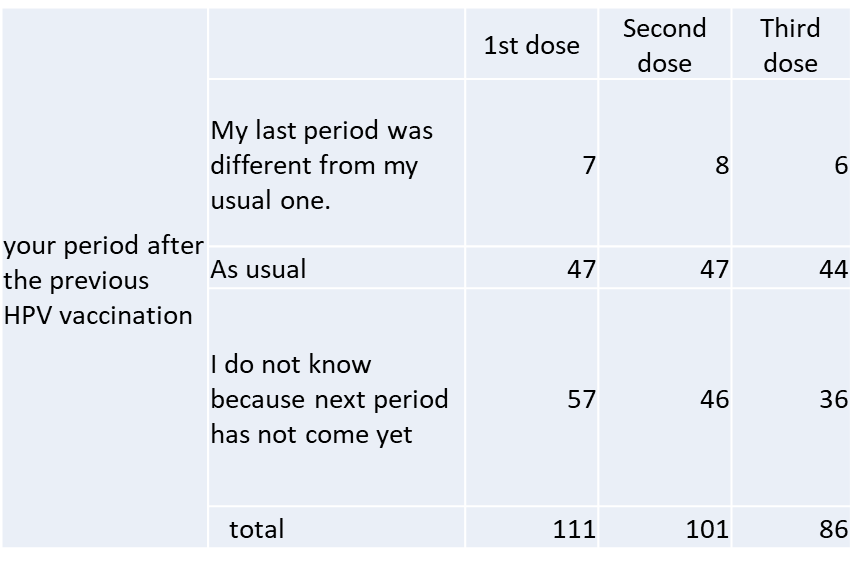


Question asked: Did the period after HPV vaccination differ from your usual period? If you were vaccinated with a second or later dose, please include the time period after the previous HPV vaccination.
